# The Effects of Fasting and Caloric Restriction on Reproductive Hormones: A Systematic Review, Meta‐Analysis, and GRADE Assessment

**DOI:** 10.1002/fsn3.72078

**Published:** 2026-07-10

**Authors:** Seyed Mojtaba Ghoreishy, Sara Rafiei, Yasamin Atabaki, Nastaran Jafarbeglou, Niloofar Hamidi, Morvarid Noormohammadi, Paniz Fatahi, Shadi Ghaemi, Farbod Shidfar, Farzad Shidfar

**Affiliations:** ^1^ School of Public Health, Iran University of Medical Sciences Tehran Iran; ^2^ Nutritional Sciences Research Center, Iran University of Medical Sciences Tehran Iran; ^3^ Department of Nutrition, School of Public Health Iran University of Medical Sciences Tehran Iran; ^4^ Department of Nutrition, Science and Research Branch Islamic Azad University Tehran Iran; ^5^ Gastroenterology and Liver Diseases Research Center, Research Institute for Gastroenterology and Liver Diseases, Shahid Beheshti University of Medical Sciences Tehran Iran; ^6^ Department of Community Nutrition School of Nutritional Sciences and Dietetics, Tehran University of Medical Sciences Tehran Iran; ^7^ School of Medicine, Iran University of Medical Sciences Tehran Iran

**Keywords:** caloric restriction, fasting, randomized controlled trials, reproductive hormones

## Abstract

Fasting and caloric restriction (CR) have shown promising effects in body composition management and metabolic health. Both CR and time‐restricted eating (TRE) have been associated with improved insulin sensitivity and lipid profiles. Although dietary and lifestyle interventions for weight management may influence reproductive hormones, the specific impact of these dietary strategies on reproductive hormones is still unclear. This systematic review and meta‐analysis assessed the effects of different types of fasting and CR on reproductive hormones in adults. Parallel or crossover randomized controlled trials published up to April 2025 were identified through PubMed, Scopus, and Google Scholar. Eligible studies included adult participants aged ≥ 18 years undergoing fasting (alternate‐day, TRE, or full‐day fasting) or CR (20%–30% energy reduction). The primary outcomes were changes in reproductive hormones, including testosterone, free testosterone, sex hormone‐binding globulin (SHBG), dehydroepiandrosterone sulfate (DHEA‐S), follicle‐stimulating hormone (FSH), luteinizing hormone (LH), estradiol, estrogen, anti‐Müllerian hormone (AMH), prolactin, and progesterone, analyzed using random‐effects models. The GRADE (Grading of Recommendations Assessment, Development, and Evaluation) approach was used to assess the certainty of evidence. Fifteen randomized trials (*n* = 954 participants) were included. Overall, both fasting and CR showed largely neutral effects on reproductive hormones, with no consistent evidence of either detrimental or beneficial changes. Fasting had a non‐significant effect on testosterone (WMD: −0.88 ng/mL; 95% CI: −2.37 to 0.61; *p* = 0.24), with significant heterogeneity. However, a statistically significant effect on free testosterone was observed (WMD: −4.9 pg/mL; 95% CI: −9.22 to −0.59; *p* = 0.02), although this finding was based on some of the small trials. Similarly, CR had a non‐significant effect on testosterone (WMD: −0.02 ng/mL; 95% CI: −0.13 to 0.08; *p* = 0.65), with significant heterogeneity. No significant effects were observed for other outcomes. Current evidence indicates that fasting and CR exert minimal to no statistically significant impact on reproductive hormones in adults. However, these findings should not be interpreted as evidence of clinical neutrality because we didn't assess changes in symptoms. Overall, these dietary strategies appear endocrinologically safe for weight management and metabolic health; however, larger, longer‐term, and population‐specific trials are needed to clarify subgroup‐specific responses.

## Graphical abstract

This graphical abstract summarizes the findings of a systematic review, meta‐analysis, and GRADE assessment evaluating the effects of fasting interventions and caloric restriction (CR) on reproductive hormones in adults. Overall, fasting and CR showed minimal to no statistically significant effects on most reproductive hormones, although fasting was associated with a significant reduction in free testosterone. These findings should be interpreted cautiously, as the available evidence is limited by small sample sizes, short intervention durations, and the absence of symptom‐based clinical outcomes.

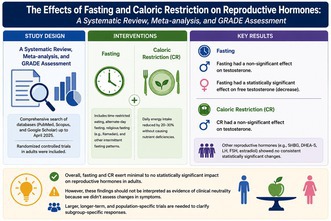

## 1 Introduction

According to recent studies, dietary interventions may potentially affect the reproductive system (Pecora et al. 2023). Because the body requires specific nutrients for the synthesis of reproductive hormones, dietary patterns may influence the normal functioning of the reproductive system and hormone levels (Pecora et al. 2023). One of the most recent dietary approaches that has gained attention regarding its effects on reproductive hormones is fasting. Fasting is a broad term, and the most common types include alternate‐day fasting (ADF) and time‐restricted eating (TRE). ADF involves alternating “feast” and “fast” days each week. TRE involves a daily eating window limited to specific hours, followed by fasting for the remaining hours (Cienfuegos et al. 2022). The most common TRE regimens include 16:8, 14:10, and 12:12, in which the eating windows are 8, 10, and 12 h, respectively (Visioli et al. 2022). Adherence to TRE may be a promising strategy for the treatment of hyperandrogenism in females with polycystic ovary syndrome (PCOS) and may also lead to reduced levels of free and total testosterone in men (Cienfuegos et al. 2022). In addition, intermittent fasting may have the potential to improve menstrual irregularities and infertility in obese women with PCOS (Mao et al. 2024). However, these dietary approaches have primarily gained attention for their effects on weight loss, insulin sensitivity, and lipid profiles (Kazeminasab et al. 2025), and evidence regarding their impact on reproductive hormones remains limited (Cienfuegos et al. 2022).

In contrast to fasting, simple caloric restriction (CR) does not involve alterations in meal timing (Chao et al. 2021; Liu et al. 2022). CR refers to a reduction in caloric intake, typically by 20%–40%, without causing macro‐ or micronutrient deficiencies (Chao et al. 2021; Duregon et al. 2021; Liu et al. 2022). Similar to fasting, CR has been associated with improvements in hyperandrogenemia, particularly through weight reduction; however, further research is needed in this area (Deshmukh et al. 2023).

Although several trials have examined the effects of fasting and CR on reproductive hormones, the findings remain inconsistent (Beitins et al. 1985; Cağlayan et al. 2014; Kalsekar et al. 2024; Mirsane et al. 2017). Furthermore, previous meta‐analyses have primarily focused on intermittent fasting and included a limited number of studies, highlighting the lack of comprehensive evidence across different fasting regimens and CR approaches (Ezzati et al. 2023). Therefore, the present meta‐analysis aimed to evaluate the effects of fasting and CR on reproductive hormones.

## 2 Methods

This systematic review and meta‐analysis employed the 2020 Preferred Reporting Items for Systematic Reviews and Meta‐analysis (PRISMA) guidelines (Page et al. 2021) to assess the effects of various types of fasting and CR on reproductive hormones (Appendix S1). The research plan was formally registered in the International Prospective Register of Systematic Reviews (PROSPERO) with the registration number CRD420251119479.

### 2.1 Search Strategy

The literature search was conducted in Scopus, PubMed/Medline, and Google Scholar databases to identify clinical trials investigating the impact of all types of fasting and CR on reproductive hormones until April 2025. Only studies published in English were included. A combination of Medical Subject Headings (MeSH) and non‐MeSH terms was used. Additionally, the gray literature search approach consisted of manually examining all original articles cited in the retrieved review articles. The search strategy is detailed in Table S1.

### 2.2 Eligibility Criteria

Studies were included if they: (1) were trials employing either crossover or parallel design, (2) involved adult participants aged over 18 years, (3) trials with a duration longer than 4 weeks, (4) included an intervention defined as CR involving a decrease in daily caloric intake of 20%–30% of energy needs, measured in kcal/day or any types of fasting, (5) provided adequate data on baseline and outcomes (testosterone, free testosterone, SHBG (sex hormone‐binding globulin), DHEA‐S (dehydroepiandrosterone sulfate), FSH (follicle‐stimulating hormone), LH (luteinizing hormone), estradiol, estrogen, AMH (anti‐Müllerian hormone), prolactin, and progesterone) in both intervention and control groups.

Articles were excluded based on the following criteria: (1) studies conducted on children, pregnant women due to the changes in reproductive hormones in this phase, or animals; (2) gray literature including conference papers, dissertations, and patents; (3) studies not classified as trials; (4) studies lacking sufficient data on the selected outcomes in either dietary intervention or control groups.

### 2.3 Study Selection

Two researchers (YA and NJ) independently screened studies that were potentially eligible. To perform a thorough evaluation, they adhered to the above‐mentioned criteria to determine which studies to include or exclude. Full texts of the selected studies were retrieved for further assessment. Any discrepancies regarding study design, methodology, or inclusion decisions were resolved by the lead researcher.

### 2.4 Data Extraction

Two reviewers (YA and NJ) independently extracted data from the included studies. The extracted characteristics of the selected studies included the first author, country, study design, population size (total and subgroup‐specific), intervention details, control conditions, and outcomes. Disagreements were resolved through discussion.

### 2.5 Risk of Bias Assessment

The quality of the study was evaluated by two independent reviewers using the modified risk of bias tool from the Cochrane Collaboration (Minozzi et al. 2020). This tool assesses several dimensions of bias, including attrition bias, performance bias, reporting bias, allocation concealment, random sequence generation, and other potential sources of bias. Every domain was classified as “low,” “high,” or “unclear” risk.

### 2.6 Quantitative Analysis

We calculated the mean changes and standard deviations (SDs) for both the diet and control groups. These values were used to estimate the total effect size as the weighted mean difference (WMD). SDs were calculated from the standard error (SE) and 95% confidence intervals (CIs), in accordance with the method described by Hozo et al. (2005). Using a random‐effects model, we pooled the unstandardized mean differences. The I‐squared (*I* ^2^) index was used to assess between‐study heterogeneity; heterogeneity was defined as “moderate” if the *I* ^2^ value was less than 50%, “substantial” if it was between 50% and 75%, and “considerable” if it was greater than 75% (Chandler et al. 2019).

Subgroup analyses were performed based on duration (≤ 16 weeks as short‐term and > 16 weeks as long‐term), location (USA vs. non‐USA), year of publication (< 2015, ≥ 2015), and sample size (< 50, ≥ 50) to explore the source of heterogeneity.

Egger's and Begg's tests were used to assess the likelihood of publication bias. A funnel plot was used to visually evaluate publication bias; a symmetric funnel‐shaped distribution indicated a low likelihood of publication bias, whereas an asymmetric distribution suggested a higher likelihood.

Furthermore, sensitivity analysis was conducted to assess the stability of the pooled effect size. All analyses were performed using Stata software (version 17), and statistical significance was defined as *p* < 0.05.

#### 2.6.1 Certainty Assessment

The GRADE working group (gradeworkinggroup.org) evaluated the overall certainty of evidence across the included studies. The quality of evidence was classified into four categories: high, moderate, low, and very low (Guyatt et al. 2008).

*Definitions of dietary interventions*:

For clarity and consistency, dietary interventions were categorized as follows:

1. Intermittent fasting (IF): eating patterns involving alternating periods of fasting and feeding, including alternate‐day fasting (ADF) and periodic fasting (e.g., 5:2 diet);
2. Time‐restricted eating (TRE): daily restriction of food intake to a specific time window (e.g., 8–10 h) without necessarily altering caloric intake;
3. Caloric restriction (CR): continuous reduction in daily energy intake without specific timing constraints;
4. Ramadan fasting: a form of religious fasting characterized by abstinence from food and drink from dawn to sunset.

## 3 Results

### 3.1 Study Selection

The search for RCTs examining the effects of all types of fasting and CR on reproductive hormones identified 5259 records, of which 1605 duplicates were removed. After screening titles and abstracts, 3603 irrelevant studies were excluded based on predefined inclusion criteria. As a result, 51 articles were selected for full‐text review. Of these articles, 36 were excluded for the following reasons (Table S2): irrelevant control group (studies were excluded when the control group included other active dietary interventions or co‐interventions that could confound the independent effects of fasting or low‐calorie diets on sex hormones) (*n* = 14 articles), short study duration (< 4 weeks) (*n* = 3 articles), no control group (*n* = 13 articles), insufficient information (*n* = 5 articles), and study protocols (*n* = 1 article). The study selection flow is presented in Figure S1.

### 3.2 Characteristics of Included Studies

Table 1 presents the baseline characteristics of the included studies. The studies were conducted between 2003 and 2025. In total, 15 RCTs and 22 study arms were included, comprising 954 participants (546 in intervention groups and 408 in control groups). Regarding sex distribution, 6 papers were done on both genders (Heilbronn et al. 2006; Lin et al. 2024; Martin et al. 2016; Moro et al. 2021; Sampieri et al. 2024; Villareal et al. 2006), 6 studies on men (Garcia‐Morales et al. 2025; Kaukua et al. 2003; Khoo et al. 2011; Moro et al. 2016, 2020; Pop et al. 2015), and 3 on women only (Kulshreshtha et al. 2024; Nybacka et al. 2013; van Gemert et al. 2015). Across all included participants, the majority were male due to the higher number of male‐only trials, whereas female‐specific data were primarily derived from studies in women with specific clinical conditions such as PCOS or postmenopausal status. Importantly, although several studies included both sexes, the majority did not report sex‐stratified outcomes, limiting the ability to perform subgroup meta‐analyses and to draw definitive conclusions regarding sex‐specific endocrine responses to fasting and CR. Five studies were conducted in the USA (Heilbronn et al. 2006; Lin et al. 2024; Martin et al. 2016; Pop et al. 2015; Villareal et al. 2006), four in Italy (Moro et al. 2016, 2020, 2021; Sampieri et al. 2024), and one each in Finland (Kaukua et al. 2003), Spain (Garcia‐Morales et al. 2025), Sweden (Nybacka et al. 2013), India (Kulshreshtha et al. 2024), Australia (Khoo et al. 2011), and the Netherlands (van Gemert et al. 2015).

**TABLE 1:** General characteristics of the effect of all types of fasting diet and calorie restricted diet on reproductive related hormones.

Author (Year), Country	Design	Health condition	Participants, *n* intervention/*n* control	Sample size (male/female)	Population sex category	Duration (week)	Groups		Mean BMI (intervention)/(control)	Mean age (intervention)/(control)	Outcomes	Results
							Intervention	Control				
Kaukua et al. (2003), Finland	Parallel	Obesity BMI ≥ 35 kg/m^2^	Middle age obese Men, 18/17	35 (35/0)	Male	10	VLED	No intervention	39.3/39.4	45.9/47.2	Testosterone/Free Testosterone/SHBG	Increase in SHBG, Testosterone and Free Testosterone
Villareal et al. (2006), USA	Parallel	Healthy	Healthy men and women, 18/9	27	Both	48	CR‐induced weight loss	Healthy lifestyle control group	27.2/27.9	55.6/56	Estradiol	No significant change
Heilbronn et al. (2006), USA	Parallel	Overweight, nonobese	Overweight, nonobese (BMI, 25–30) men and women, 12/12	24 (11/13)	Both	24	CR (25% calorie restriction of baseline energy requirements)	Weight maintenance diet	27.8/27.8	39/37	DHEA‐S	No significant change
Khoo et al. (2011), Australia	Parallel	Obesity, Type 2 diabetes	Obese type 2 diabetic men, 9/7	16 (16/0)	Male	52	LCD	High‐protein, Low‐fat Diet	35.1/35.6	58.1/62.3	Testosterone/SHBG	Increase in SHBG
Nybacka et al. (2013), Sweden	Parallel	Obesity PCOS	Obese women with PCOS, 12/17	29 (0/29)	Female	16	Diet and exercise	Exercise	38.1/34.8	31.8/31.3	Testosterone/FSH/LH/Free Testosterone/SHBG/AMH	Reduction in AMH
van Gemert et al. (2015), Netherland	Parallel	Overweight	Postmenopausal women, 97/48	145 (0.145)	Female	16	CR	Stable weight control group	29.3/29.5	60.5/60	Testosterone/Free Testosterone/Estradiol/Free Estradiol/SHBG	Reduction in Estradiol and Free Estradiol
							Weight loss diet					Increase in SHBG
Pop et al. (2015), USA	Parallel	Obesity and overweight	Obese and overweight men, 19/19	38 (38/0)	Male	24	Weight loss through CR	Weight maintenance	32.4/30.6	57.7/59.7	Testosterone/Free Testosterone/Estradiol/Free Estradiol/SHBG	Reduction in Estradiol and Free Estradiol
Moro et al. (2016), Italy	Parallel	Healthy	Healthy resistance‐trained males, 17/17	34 (34/0)	Male	8	TRE (16/8)	ND		29.94/28.47	Testosterone	Reduction in Testosterone
Martin et al. (2016), USA	Parallel	Healthy	Healthy Nonobese Adults, 143/75	218 (66/152)	Both	96	CR	Ad libitum	25.2/25.1	38/37.9	Testosterone/Free Testosterone/FSH/LH/SHBG	Increase in SHBG
Moro et al. (2020), Italy	Parallel	Healthy	Healthy young men, 8/8	16 (16/0)	Male	4	TRE (16/8)	ND	21.85/22.47	19.38/19.38	Free Testosterone/SHBG	Reduction in Free Testosterone
Moro et al. (2021), Italy	Parallel	Healthy	Healthy subjects, 10/10	20	Both	52	TRE (16/8)	ND			Testosterone	Reduction in Testosterone
Kulshreshtha et al. (2024), India	Parallel	PCOS	PCOS women, 69/65	134 (0/134)	Female	12	Hypocaloric diet	Isocaloric diet	26.38/25.87	22.88/24.74	Testosterone/Free Testosterone/LH/FSH/SHBG/Prolactin	
Sampieri et al. (2024), Italy	Parallel	Healthy	Healthy, non‐trained individuals 10/10	20 (11/9)	Both	8	TRE (16/8)	ND	26.78/24.54	41.5/41.7	Free Testosterone	
Sampieri et al. (2024), Italy	Parallel	Healthy	Healthy, non‐trained individuals 10/10	20 (10/10)	Both	8	TRE (14:10)	ND	24.95/24.54	44.4/41.7	Free Testosterone	
Sampieri et al. (2024), Italy	Parallel	Healthy	Healthy, non‐trained individuals 10/10	20 (11/9)	Both	8	TRE (12:12)	ND	24.67/24.54	38.1/41.7	Free Testosterone	
Lin et al. (2024), USA	Parallel	Obesity	Males (5/5)	10	Male	52	TRE (16/8)	ND	30–50	18–65	Testosterone, DHEA, SHBG	No significant change
Lin et al. (2024), USA	Parallel	Obesity	Males (5/5)	10	Male	52	CR	ND	30–50	18–65	Testosterone, DHEA, SHBG	No significant change
Lin et al. (2024), USA	Parallel	Obesity	Premenopausal females (22/22)	44	Female	52	TRE (16/8)	ND	30–50	18–65	Testosterone, DHEA, SHBG	No significant change
Lin et al. (2024), USA	Parallel	Obesity	Premenopausal females (22/22)	44	Female	52	CR	ND	30–50	18–65	Testosterone, DHEA, SHBG	No significant change
Lin et al. (2024), USA	Parallel	Obesity	Postmenopausal females (9/10)	19	Female	52	TRE (16/8)	ND	30–50	18–65	Testosterone, DHEA, SHBG, Estradiol, Estrogen	No significant change
Lin et al. (2024), USA	Parallel	Obesity	Postmenopausal females (9/10)	19	Female	52	CR	ND	30–50	18–65	Testosterone, DHEA, SHBG, Estradiol, Estrogen	No significant change
Garcia‐Morales et al. (2025), Spain	Parallel	Healthy	Male professional soccer players, 12/16	28 (28/0)	Male	12	CR	ND		27.8/27.5	Testosterone	No significant change

Abbreviations: ADF: alternate‐day fasting; CR: Caloric restriction; IF: Intermittent fasting; LCD: Low‐energy Diet; ND: Normal diet; TRE: Time‐restricted eating; VLED: A very‐low‐energy diet.

The intervention duration ranged from 4 to 96 weeks. All studies had a parallel study design. Five studies (Heilbronn et al. 2006; Kaukua et al. 2003; Lin et al. 2024; Pop et al. 2015; van Gemert et al. 2015), were conducted in overweight or obese participants, two in patients with PCOS (Kulshreshtha et al. 2024; Nybacka et al. 2013), seven in healthy participants (Garcia‐Morales et al. 2025; Martin et al. 2016; Moro et al. 2016, 2020, 2021; Sampieri et al. 2024; Villareal et al. 2006) and one in patients with diabetes (Khoo et al. 2011). Two studies (Lin et al. 2024; Sampieri et al. 2024) included more than one intervention group. Among the 15 studies (22 intervention arms), 13 were related to low‐calorie diets, and the remaining arms involved various types of fasting. A summary of the evidence is presented in Table 2. Only 3 of the 15 trials were judged to have a low risk of bias, while the remaining 12 were assessed as high risk of bias (Table S4).

**TABLE 2:** The effects of all types of fasting diet and calorie restricted diet on reproductive related hormones.

Outcomes	Number of trials (arms), participants	Type of effect size	Effect size (95% CI)	GRADE certainty
Testosterone (Studies on fasting diet)	3 (5), 127	Mean difference	−0.88 ng/mL (−2.37 to 0.61)	Very low
Testosterone (Studies on calorie restricted diet)	9 (11), 716	Mean difference	−0.02 ng/mL (−0.13 to 0.08)	Very low
Free Testosterone (Studies on fasting diet)	2 (4), 76	Mean difference	−4.9 pg/mL (−9.22 to −0.59)	Moderate
Free Testosterone (Studies on calorie restricted diet)	6 (6), 599	Mean difference	0.21 pg/mL (−2.65 to 3.06)	Very low
SHBG (Studies on fasting diet)	2 (4), 89	Mean difference	−3.87 ng/mL (−103.81 to 96.07)	Very low
SHBG (Studies on calorie restricted diet)	8 (10), 688	Mean difference	43.97 ng/mL (−95.15 to 183.08)	Very low
DHEA‐S (Studies on fasting diet)	1 (3), 73	Mean difference	−0.18 ng/mL (−0.68 to 0.31)	Very low
DHEA‐S (Studies on calorie restricted diet)	2 (4), 97	Mean difference	−0.11 ng/mL (−0.41 to 0.20)	Very low
LH (Studies on calorie restricted diet)	3 (3), 381	Mean difference	0.03 mIU/mL (−1.17 to 1.22)	Very low
FSH (Studies on calorie restricted diet)	3 (3), 381	Mean difference	0.17 mIU/mL (−0.28 to 0.62)	Low
Estradiol (Studies on calorie restricted diet)	4 (4), 205	Mean difference	0.08 pg/mL (−7.31 to 7.47)	Very low
Estradiol (Studies on fasting diet)	1 (1), 19	Mean difference	−0.93 pg/mL (−2.19, 0.33)	—
Estrogen (Studies on fasting diet)	1 (1), 19	Mean difference	−11.98 pg/mL (−14.92, −9.03)	—
Estrogen (Studies on calorie restricted diet)	1 (1), 19	Mean difference	−12.73 pg/mL (−15.74, −9.71)	—
AMH (Studies on calorie restricted diet)	1 (1), 29	Mean difference	−10.00 pmol/L (−23.75, 3.75)	—
Prolactin (Studies on calorie restricted diet)	1 (1), 134	Mean difference	1.79 ng/mL (0.009, 3.57)	—
Progesterone (Studies on fasting diet)	1 (1), 19	Mean difference	0.02 ng/mL (−0.01, 0.05)	—
Progesterone (Studies on calorie restricted diet)	1 (1), 19	Mean difference	−0.15 ng/mL (−0.18, −0.11)	—

Abbreviations: CI, confidence interval; GRADE, Grading of Recommendations, Assessment, Development, and Evaluation.

### 3.3 Meta‐Analysis Results

#### 3.3.1 Effects of Fasting and CR on Testosterone

##### 3.3.1.1 Fasting

**FIGURE 1:**
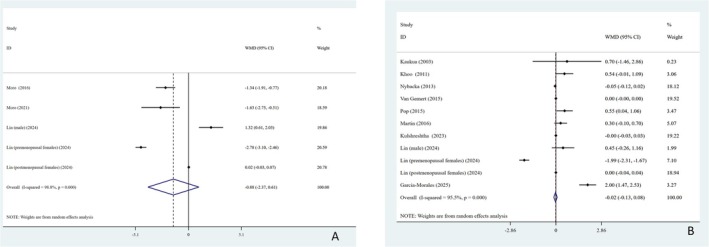
Forest plot for the effect of all types of fasting and caloric restriction on testosterone expressed as mean difference between intervention and control groups. The area of each square is proportional to the inverse of the variance of the WMD. Horizontal lines represent 95% CIs. Diamonds represent pooled estimates from random effects analysis. WMD, weighted mean difference. (A) Studies on fasting; (B) Studies on caloric restriction.

Overall, 5 effect sizes from 3 studies, with a total sample size of 127 adults, were included in the analysis of the effect of fasting on testosterone. Pooling these effect sizes, we found a non‐significant effect of fasting on testosterone [weighted mean difference (WMD): −0.88; 95% CI: −2.37 to 0.61 ng/mL; *p* = 0.24] (Figure 1A).

A significant level of heterogeneity was detected among studies (*I* ^2^ = 98.8%, *p* < 0.001). Subgroup analysis suggested that study location may explain the between‐study heterogeneity (Table S3). Sensitivity analysis showed that the overall estimate was not dependent on any single study. No evidence of publication bias was observed based on Egger's test (*p* = 0.36).

##### 3.3.1.2 Caloric Restriction, CR

Overall, 11 effect sizes from 9 studies, with a total sample size of 716 adults, were included in the analysis of the effect of CR on testosterone. Pooling these effect sizes, we found a non‐significant effect of CR on testosterone [WMD: −0.02; 95% CI: −0.13 to 0.08 ng/mL; *p* = 0.65] (Figure 1B). A significant level of heterogeneity was detected among studies (*I* ^2^ = 95.5%, *p* < 0.001). Despite subgroup analyses, no clear source of heterogeneity was identified, and results remained non‐significant across all categories (Table S3). Sensitivity analysis indicated that the overall estimate was not driven by any single study. No evidence of publication bias was observed based on Egger's test (*p* = 0.95).

#### 3.3.2 Effect of Fasting and CR on Free Testosterone

##### 3.3.2.1 Fasting

**FIGURE 2:**
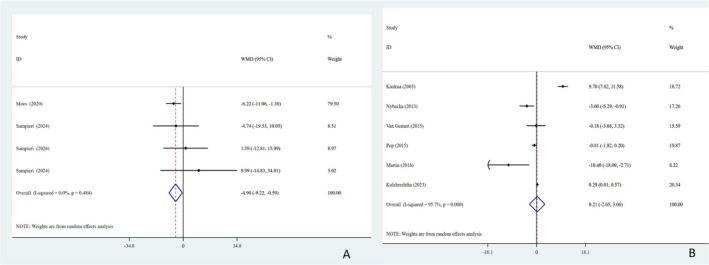
Forest plot for the effect of all types of fasting and caloric restriction on free testosterone expressed as mean difference between intervention and control groups. The area of each square is proportional to the inverse of the variance of the WMD. Horizontal lines represent 95% CIs. Diamonds represent pooled estimates from random effects analysis. WMD, weighted mean difference. (A) studies on fasting; (B) studies on caloric restriction.

Pooling 4 effect sizes from 2 studies, with a total sample size of 76 adults, showed a significant effect of fasting on free testosterone [WMD: −4.9; 95% CI: −9.22, −0.59 pg/mL; *p* = 0.02] (Figure 2A). Sensitivity analysis indicated that the exclusion of any single study did not materially change the overall findings. No evidence of publication bias was detected based on Egger's test (*p* = 0.11).

##### 3.3.2.2 Caloric Restriction, CR

Pooling effect sizes from 6 studies with a total sample size of 599 adults, we found a non‐significant effect of CR on free testosterone [WMD: 0.21; 95% CI: −2.65, 3.06 pg/mL; *p* = 0.56] (Figure 2B).

A significant level of heterogeneity was detected among studies (*I* ^2^ = 95.7%, *p* < 0.001). Subgroup analyses suggested that the year of publication, location, and sample size may explain the between‐study heterogeneity (Table S3). Notably, in studies with a sample size of fewer than 50 participants, free testosterone levels increased significantly [WMD: 1.03; 95% CI: 0.18, 1.87 pg/mL].

Sensitivity analysis showed that exclusion of any single study did not materially affect the overall findings. No evidence of publication bias was observed based on Egger's test (*p* = 0.91).

#### 3.3.3 Effect of Fasting and CR on SHBG

##### 3.3.3.1 Fasting

**FIGURE 3:**
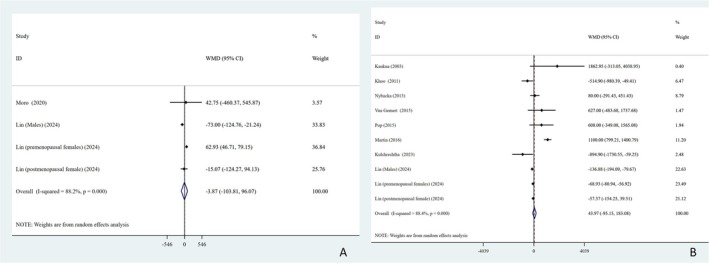
Forest plot for the effect of all types of fasting and caloric restriction on SHBG expressed as mean difference between intervention and control groups. The area of each square is proportional to the inverse of the variance of the WMD. Horizontal lines represent 95% CIs. Diamonds represent pooled estimates from random effects analysis. WMD, weighted mean difference. (A) Studies on fasting; (B) Studies on caloric restriction.

A total of 2 studies providing 4 effect sizes, with a total sample size of 89 individuals, were included in the analysis of the effect of fasting on SHBG. Pooling these effect sizes, we found a non‐significant effect of fasting on SHBG [WMD: −3.87; 95% CI: −103.81 to 96.07 ng/mL; *p* = 0.94] (Figure 3A), with significant between‐study heterogeneity (*I* ^2^ = 88.2%, *p* < 0.001).

Subgroup analysis suggested that study location may explain the observed heterogeneity (Table S3).

Sensitivity analysis indicated that the overall effect size was not influenced by any single study. No significant evidence of publication bias was observed based on Egger's test (*p* = 0.41).

##### 3.3.3.2 Caloric Restriction, CR

A total of 8 studies providing 10 effect sizes, with a total sample size of 688 individuals, were included in the analysis of the effect of CR on SHBG. Pooling these effect sizes, we found a non‐significant effect of CR on SHBG [WMD: 43.97; 95% CI: −95.15 to 183.08 ng/mL; *p* = 0.53] (Figure 3B), with significant between‐study heterogeneity (*I* ^2^ = 88.4%, *p* < 0.001).

Subgroup analyses suggested that year of publication, location, and intervention duration may explain the between‐study heterogeneity (Table S3). Sensitivity analysis showed that the overall effect size was not driven by any single study. No significant evidence of publication bias was observed based on Egger's test (*p* = 0.42).

#### 3.3.4 Effects of Fasting and CR on DHEA‐S

##### 3.3.4.1 Fasting

**FIGURE 4:**
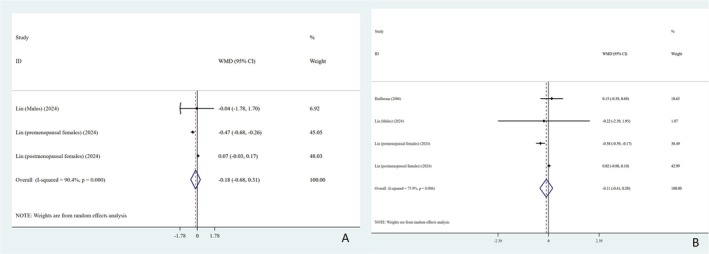
Forest plot for the effect of all types of fasting and caloric restriction on DHEA‐S expressed as mean difference between intervention and control groups. The area of each square is proportional to the inverse of the variance of the WMD. Horizontal lines represent 95% CIs. Diamonds represent pooled estimates from random effects analysis. WMD, weighted mean difference. (A) Studies on fasting; (B) Studies on caloric restriction.

Three effect sizes with a total sample size of 73 adults were included in this analysis. Pooling these effect sizes, we found no significant effect of fasting on DHEA‐S [WMD: −0.18; 95% CI: −0.68 to 0.31 ng/mL; *p* = 0.47] (Figure 4A).

A significant level of heterogeneity was detected among studies (*I* ^2^ = 90.4%, *p* < 0.001). Due to the small number of studies, subgroup analysis could not be performed to identify sources of heterogeneity. No significant evidence of publication bias was observed based on Egger's test (*p* = 0.70).

##### 3.3.4.2 Caloric Restriction, CR

Four effect sizes with a total sample size of 97 adults were included in this analysis. Pooling these effect sizes, we found no significant effect of CR on DHEA‐S [WMD: −0.11; 95% CI: −0.41 to 0.20 ng/mL; *p* = 0.49] (Figure 4B).

A significant level of heterogeneity was detected among studies (*I* ^2^ = 75.9%, *p* = 0.006). Due to the small number of studies, subgroup analysis was not performed. Sensitivity analysis indicated that the overall effect size was not influenced by any single study. No significant evidence of publication bias was observed based on Egger's test (*p* = 0.65, Egger's test).

#### 3.3.5 Effects of CR on LH

**FIGURE 5:**
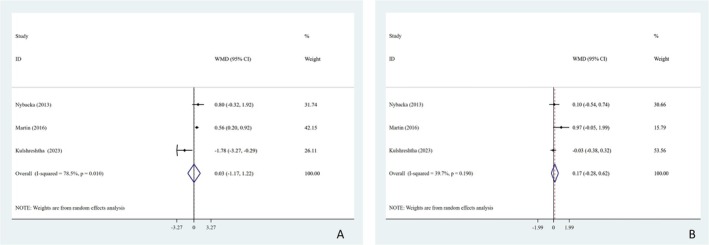
Forest plot for the effect of all types of fasting and caloric restriction on LH and FSH expressed as mean difference between intervention and control groups. The area of each square is proportional to the inverse of the variance of the WMD. Horizontal lines represent 95% CIs. Diamonds represent pooled estimates from random effects analysis. WMD, weighted mean difference. (A) The effect of all types of caloric restriction on LH. (B) The effect of all types of caloric restriction on FSH.

Combining 3 effect sizes with a total sample size of 381 individuals showed no significant effect of CR on LH [WMD: 0.03 mIU/mL; 95% CI: −1.17 to 1.22; *p* = 0.96] (Figure 5A). Significant between‐study heterogeneity was observed (*I* ^2^ = 78.5%, *p* = 0.01). Subgroup analyses suggested that year of publication, location, and sample size may explain the heterogeneity (Table S3).

Sensitivity analysis indicated that the exclusion of Kulshreshtha et al. (2024) study materially changed the results (WMD: 0.58, 95% CI: 0.23 to 0.92). No evidence of publication bias was observed (*p* = 0.56, Egger's test).

#### 3.3.6 Effects of CR on FSH

Combining 3 effect sizes with a total sample size of 381 individuals showed no significant effect of CR on FSH [WMD: 0.17 mIU/mL; 95% CI: −0.28 to 0.62; *p* = 0.46] (Figure 5B).

No significant between‐study heterogeneity was observed (*I* ^2^ = 39.7%, *p* = 0.19). Sensitivity analysis indicated that the overall effect size was not influenced by any single study. No evidence of publication bias was observed (*p* = 0.26, Egger's test).

#### 3.3.7 Effects of CR on Estradiol

**FIGURE 6:**
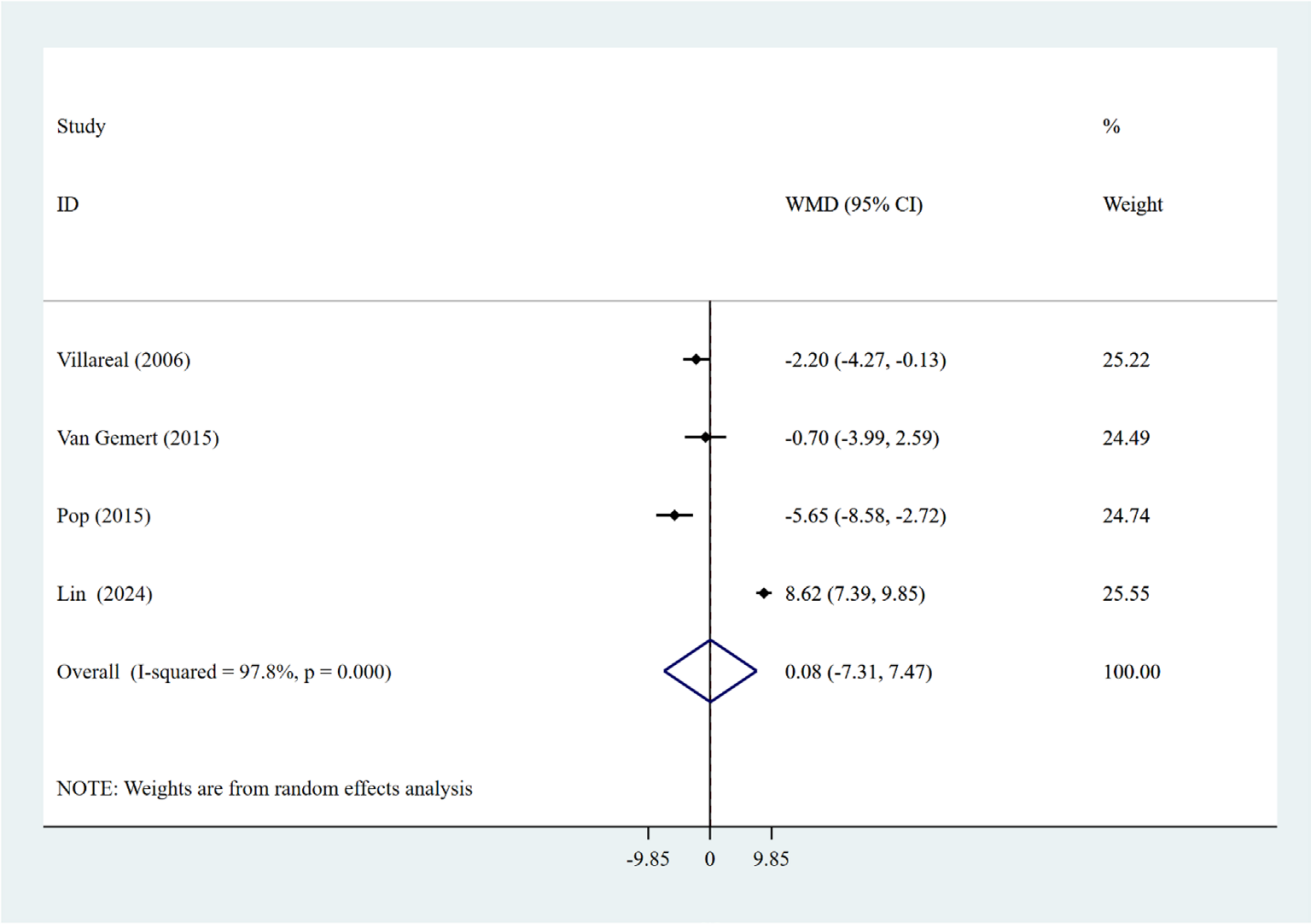
Forest plot for the effect of all types of caloric restriction on estradiol expressed as mean difference between intervention and control groups. The area of each square is proportional to the inverse of the variance of the WMD. Horizontal lines represent 95% CIs. Diamonds represent pooled estimates from random effects analysis. WMD, weighted mean difference.

Four studies providing 4 effect sizes, with a total sample size of 205 individuals, were included in this analysis. Pooling these effect sizes showed no significant effect of CR on estradiol [WMD: 0.08 pg/mL; 95% CI: −7.31 to 7.47; *p* = 0.98] (Figure 6).

Significant heterogeneity was observed among studies (*I* ^2^ = 97.8%, *p* < 0.001). Subgroup analyses suggested that location and sample size may explain the heterogeneity (Table S3).

Sensitivity analysis indicated that exclusion of the Lin et al. (2024) study materially changed the results (WMD: 2.86 pg/mL, 95% CI: −5.48 to −0.24). No evidence of publication bias was observed based on Egger's test (*p* = 0.11).

Due to the small number of studies available for several outcomes, including estrogen (fasting, CR), estradiol (fasting), free estradiol (fasting, CR), AMH (fasting, CR), prolactin (fasting, CR), and progesterone (fasting, CR), these variables were not included in the meta‐analysis and were instead evaluated qualitatively in the systematic review.

### 3.4 Grading the Evidence

The certainty of evidence was rated using the GRADE rating tool (Table S5). Testosterone (Studies on fasting), Testosterone (Studies on CR), Free Testosterone (Studies on caloric restriction), SHBG (Studies on fasting), SHBG (Studies on CR), DHEA‐S (Studies on fasting), DHEA‐S (Studies on CR), LH (Studies on CR), Estradiol (Studies on CR) had a very low evidence rating; on the other hand, FSH (Studies on CR) had a low evidence rating and Free Testosterone (Studies on fasting) had a moderate evidence rating.

Given the heterogeneity in sex distribution across studies, interpretation of pooled hormonal outcomes should be made with caution. Testosterone outcomes were predominantly derived from male populations, whereas data on gonadotropins and estradiol were largely driven by female‐specific studies. This imbalance may partially explain variability in effect estimates and limit the generalizability of findings across sexes.

## 4 Discussion

This systematic review and meta‐analysis provide the most comprehensive synthesis to date on the effects of fasting and CR on reproductive hormones in adults. Overall, both dietary strategies exerted neutral to modest effects, with no consistent detrimental or beneficial impact on testosterone, estradiol, SHBG, LH, FSH, or DHEA‐S. Only free testosterone showed a significant decrease under fasting interventions; however, this finding was based on a limited number of small trials and should be interpreted with caution. Collectively, the evidence suggests that energy restriction—whether through intermittent fasting or CR—is unlikely to exert clinically meaningful adverse effects on reproductive endocrine function in the general adult population.

Sex‐specific physiological differences may modulate endocrine responses to energy restriction. While male participants generally exhibited stable testosterone levels, studies in women, particularly those with PCOS or postmenopausal status, demonstrated greater variability in gonadotropins and estradiol. However, the limited number of sex‐stratified analyses precluded robust subgroup meta‐analysis, highlighting an important direction for future research.

These findings build on and extend prior work by broadening both the populations and the hormonal outcomes assessed. Earlier evidence syntheses, particularly in women with PCOS, focused narrowly on intermittent fasting and a limited set of hormones, relying on a small number of often underpowered trials (16). By contrast, the present analysis incorporated a larger and more diverse body of RCTs, encompassing both fasting and CR, and evaluated a wider panel of sex hormones. This expanded scope enables a more generalizable characterization of endocrine responses across varied populations and dietary contexts.

Sensitivity analyses revealed that select context‐specific trials disproportionately influenced pooled estimates, highlighting the role of population characteristics and study design in driving heterogeneity. For example, in the subgroup of postmenopausal women, Lin et al. (Lin et al. 2024) reported modest reductions in estradiol levels with TRE, suggesting that menopausal status may modify endocrine responses. However, this long‐duration trial included both sexes with obesity and assessed reproductive hormones as secondary endpoints, which limited comparability due to the small subgroup sizes.

Similarly, in women with PCOS, gonadotropin alterations were primarily driven by the trial of Kulshreshtha et al. (Kulshreshtha et al. 2024), which differed from most other studies by comparing isocaloric with hypocaloric diets, assessing a broad hormonal panel, and permitting pharmacologic therapy when lifestyle modification alone was insufficient. These methodological differences complicate attribution of hormonal changes solely to dietary intervention and distinguish these studies from diet‐only RCTs included in this review.

Compared with the predominantly short‐term, small‐sample trials conducted in healthy or overweight adults, the distinct design and specialized populations of these longer‐duration studies help explain their disproportionate influence on the overall synthesis. Taken together, these observations suggest that variability in hormonal outcomes reflects not only sample size but also inherent differences in study populations and intervention designs, underscoring the importance of contextual interpretation of pooled results.

From a biological perspective, the modest hormonal shifts observed are plausible. Moderate energy restriction improves insulin sensitivity and reduces adiposity without substantially disrupting.

Hypothalamic–pituitary–gonadal axis activity, thereby preserving GnRH pulsatility and downstream hormone production (Hoffer et al. 1986). Improved insulin sensitivity may enhance hepatic SHBG synthesis (O'Connor et al. 2010), while reduced adiposity may limit aromatase‐mediated estrogen production (Pop et al. 2015; Siiteri 1987; Simpson 2003; van Gemert et al. 2015). These mechanisms likely explain the subtle hormonal changes observed in specific subgroups.

Additionally, intervention duration appears to be a critical factor. Short‐term fasting trials (Moro et al. 2016, 2020, 2021) reported transient reductions in testosterone, whereas longer‐term interventions (Lin et al. 2024; Martin et al. 2016) demonstrated hormonal normalization, consistent with adaptive endocrine responses.

To our knowledge, this is the first meta‐analysis to systematically evaluate the effects of both fasting and CR on reproductive hormones in adults, employing rigorous methodology that includes preregistration, adherence to PRISMA 2020 guidelines, comprehensive searches across multiple databases, and GRADE‐based certainty assessment.

However, several limitations should be acknowledged. Only a limited number of trials assessed AMH, prolactin, or progesterone. Considerable heterogeneity existed across interventions and populations, complicating interpretation, and most studies were short‐term, leaving the long‐term effects uncertain. Assay variability required unit conversions, which may have introduced minor errors. Additionally, the certainty of evidence was rated as very low for most outcomes.

From a clinical perspective, based on the available evidence, intermittent fasting and CR appear safe and effective for weight management, with minimal impact on reproductive hormones and no evidence of adverse effects on fertility. These findings support the use of dietary restriction as a viable weight management strategy that does not compromise reproductive health. Contextual factors, such as PCOS or postmenopausal status, may influence specific hormonal responses; however, overall, these diets can be considered endocrinologically safe for most populations.

Looking ahead, future research should focus on larger, longer‐duration, and better‐stratified randomized trials with standardized hormone assays and habitual diet comparators to isolate fasting‐specific effects. Mechanistic studies are also needed to elucidate the interplay between insulin sensitivity, adiposity, SHBG production, and hypothalamic regulation, which may explain the variability observed across populations.

## 5 Conclusions

In summary, although both fasting and CR appear endocrinologically safe for most adults, and no clinically meaningful adverse effects on key reproductive hormones were observed, fasting and CR do not exert clinically meaningful adverse effects on sex hormones in adults. Across randomized trials, testosterone, estradiol, SHBG, LH, FSH, and DHEA‐S remained largely unchanged, with only free testosterone showing a modest decrease under fasting conditions in a limited number of studies. These findings support the endocrine safety of caloric restriction and affirm its role as a safe and effective approach to weight management and metabolic health in adults.

## Author Contributions

**Nastaran Jafarbeglou:** data curation. **Shadi Ghaemi:** writing – review and editing. **Seyed Mojtaba Ghoreishy:** conceptualization, writing – review and editing, formal analysis. **Sara Rafiei:** data curation, investigation, writing – review and editing. **Paniz Fatahi:** data curation. **Morvarid Noormohammadi:** writing – review and editing, conceptualization. **Niloofar Hamidi:** writing – review and editing. **Farbod Shidfar:** writing – review and editing. **Yasamin Atabaki:** data curation. **Farzad Shidfar:** supervision, writing – review and editing.

## Funding

This study was financially supported by a grant from the student research committee, School of Public Health, Iran University of Medical Sciences, Tehran, Iran.

## Ethics Statement

The authors have nothing to report.

## Consent

The data provided to the researchers did not include any personal information, and all participants were adults. Not applicable.

## Conflicts of Interest

The authors declare no conflicts of interest.

## Supporting information


**Data S1:** PRISMA checklist.

**Table S1:** Search strategies including the key terms and the queries for each database.
**Table S2:** Reason for exclusion of retrieved articles.
**Table S3:** Subgroup analysis to evaluate the effect of all types of fasting and caloric restriction on reproductive hormones.
**Table S4:** Risk of bias of included studies.
**Table S5:** GRADE evidence table for the effects of fasting and caloric restriction on reproductive hormones.
**Figure S1:** Flow diagram of study.

## Data Availability

All data analyzed in this study are derived from previously published studies and are available within the cited articles and Supporting Information. No new datasets were generated.
