# Supplementary material for: The Effects of Fasting and Caloric Restriction on Reproductive Hormones: A Systematic Review, Meta‐Analysis, and GRADE Assessment
Source: Food Sci Nutr. 2026 Jul 10;14(7):e72078. doi: 10.1002/fsn3.72078 (PMC13352353; doi:10.1002/fsn3.72078)
Supplement: Supplementary file 2 — Table S1: Search strategies including the key terms and the queries for each database. Table S2: Reason for exclusion of retrieved articles. Table S3: Subgroup analysis to evaluate the effect of all types of fasting and caloric restriction on reproductive hormones. Table S4: Risk of bias of included studies. Table S5: GRADE evidence table for the effects of fasting and caloric restriction on reproductive hormones. Figure S1: Flow diagram of study. [file FSN3-14-e72078-s002.docx]

**The effect of all types of fasting diet and calorie restricted diet on reproductive related hormones: A systematic review, meta-analysis, and GRADE assessment**

**Supplementary Tables 1-5 and Supplementary Figures 1**

| **Supplementary Table 1.** Search strategies including the key terms and the queries for each database. **4/13/2025** | |
| --- | --- |
| **Database** | **key terms and the queries** |
| PubMed  (2,003) | **#1** ("IF"[Title/Abstract] OR "energy restriction"[Title/Abstract] OR FMD "Intermittent Fasting"[Title/Abstract] OR "Energy-Restricted Diet"[Title/Abstract] OR "fasting"[Title/Abstract] OR "intermittent* fast*"[Title/Abstract] OR "fast* diet*"[Title/Abstract] OR "modified fast*"[Title/Abstract] OR "food abstinence"[Title/Abstract] OR "food fast*"[Title/Abstract] OR "food restricti*"[Title/Abstract] OR "time restricted feed*"[Title/Abstract] OR "time restricted fast*"[Title/Abstract] OR "time-restricted eating"[Title/Abstract] OR "food tim*"[Title/Abstract] OR "recurrent circadian fasting"[Title/Abstract] OR "intermittent caloric restriction"[Title/Abstract] OR "alternate day fasting"[Title/Abstract] OR "5:2 diet"[Title/Abstract] OR "Alternate-Day Fasting"[Title/Abstract] OR "meal timing"[Title/Abstract] OR "meal frequency"[Title/Abstract] OR "delayed meal"[Title/Abstract] OR Starvation[Title/Abstract] OR "low energy"[Title/Abstract] OR "low-energy"[Title/Abstract] OR "fasting-mimicking diets"[Title/Abstract] OR "Caloric Restriction"[Title/Abstract]) OR (((("Intermittent Fasting"[Mesh]) OR "Fasting"[Mesh]) OR "Caloric Restriction"[Mesh]) OR (("Starvation"[Mesh]) OR "Caloric Restriction"[Mesh]))  **#2** (Testosterone[Title/Abstract] OR "Anti-Mullerian Hormone"[Title/Abstract] OR "Free androgen index"[Title/Abstract] OR Progesterone[Title/Abstract] OR Estrogen[Title/Abstract] OR "Follicle Stimulating Hormone"[Title/Abstract] OR "FSH"[Title/Abstract] OR "Luteinising hormone"[Title/Abstract] OR "LH"[Title/Abstract] OR "Gonadotropin releasing hormone"[Title/Abstract] OR "GnRH"[Title/Abstract] OR Androgens[Title/Abstract] OR Inhibin[Title/Abstract] OR Prolactin[Title/Abstract] OR "Reproductive hormones"[Title/Abstract] OR Estradiol[Title/Abstract] OR "Sex Hormones"[Title/Abstract] OR DHEA[Title/Abstract]) OR (((((((((((("Testosterone"[Mesh]) OR "Anti-Mullerian Hormone"[Mesh]) OR "Progesterone"[Mesh]) OR "Estrogens"[Mesh]) OR "Follicle Stimulating Hormone"[Mesh]) OR "Luteinizing Hormone"[Mesh]) OR "Gonadotropin-Releasing Hormone"[Mesh]) OR "Androgens"[Mesh]) OR "Inhibins"[Mesh]) OR "Estradiol"[Mesh]) OR "Gonadal Steroid Hormones"[Mesh]) OR "Dehydroepiandrosterone"[Mesh])  **#3**(intervention[tiab] OR RCT[tiab] OR "controlled trial"[tiab] OR randomized[tiab] OR random[tiab] OR Randomly[tiab] OR Placebo[tiab] OR Assignment[tiab] OR "clinical trial"[tiab] OR trial[tiab] OR randomised[tiab] OR "Methods"[Mesh] OR "Randomized Controlled Trial"[Publication Type] OR "Controlled Clinical Trial"[Publication Type] OR "Placebos"[Mesh] OR "Placebo Effect"[Mesh] OR "Clinical Trial"[Publication Type] OR "Clinical Trials as Topic"[Mesh])  **#1 and #2 and #3** |
| Scopus  (3,256) | **#1** ( TITLE-ABS-KEY ( if ) OR TITLE-ABS-KEY ( "energy restriction" ) OR TITLE-ABS-KEY ( fmd ) OR TITLE-ABS-KEY ( "Intermittent Fasting" ) OR TITLE-ABS-KEY ( "Energy-Restricted Diet" ) OR TITLE-ABS-KEY ( "fasting" ) OR TITLE-ABS-KEY ( "intermittent* fast*" ) OR TITLE-ABS-KEY ( "fast* diet*" ) OR TITLE-ABS-KEY ( "modified fast*" ) OR TITLE-ABS-KEY ( "food abstinence" ) OR TITLE-ABS-KEY ( "food fast*" ) OR TITLE-ABS-KEY ( "food restricti*" ) OR TITLE-ABS-KEY ( "time restricted feed*" ) OR TITLE-ABS-KEY ( "time restricted fast*" ) OR TITLE-ABS-KEY ( "time-restricted eating" ) OR TITLE-ABS-KEY ( "food tim*" ) OR TITLE-ABS-KEY ( "recurrent circadian fasting" ) OR TITLE-ABS-KEY ( "intermittent caloric restriction" ) OR TITLE-ABS-KEY ( "alternate day fasting" ) OR TITLE-ABS-KEY ( "5:2 diet" ) OR TITLE-ABS-KEY ( "Alternate-Day Fasting" ) OR TITLE-ABS-KEY ( "meal timing" ) OR TITLE-ABS-KEY ( "meal frequency" ) OR TITLE-ABS-KEY ( "delayed meal" ) OR TITLE-ABS-KEY ( starvation ) OR TITLE-ABS-KEY ( "low energy" ) OR TITLE-ABS-KEY ( "low-energy" ) OR TITLE-ABS-KEY ( "fasting-mimicking diets" ) OR TITLE-ABS-KEY ( "Caloric Restriction" ) )  **#2**( TITLE-ABS-KEY ( testosterone ) OR TITLE-ABS-KEY ( "Anti-Mullerian Hormone" ) OR TITLE-ABS-KEY ( "Free androgen index" ) OR TITLE-ABS-KEY ( progesterone ) OR TITLE-ABS-KEY ( estrogen ) OR TITLE-ABS-KEY ( "Follicle Stimulating Hormone" ) OR TITLE-ABS-KEY ( "FSH" ) OR TITLE-ABS-KEY ( "Luteinising hormone" ) OR TITLE-ABS-KEY ( lh ) OR TITLE-ABS-KEY ( "Gonadotropin releasing hormone" ) OR TITLE-ABS-KEY ( gnrh ) OR TITLE-ABS-KEY ( androgens ) OR TITLE-ABS-KEY ( inhibin ) OR TITLE-ABS-KEY ( prolactin ) OR TITLE-ABS-KEY ( "Reproductive hormones" ) OR TITLE-ABS-KEY ( estradiol ) OR TITLE-ABS-KEY ( "Sex Hormones" ) OR TITLE-ABS-KEY ( dhea ) )  **#3** ( TITLE-ABS-KEY ( intervention )  OR  TITLE-ABS-KEY ( RCT )  OR  TITLE-ABS-KEY ( "controlled trial" )  OR  TITLE-ABS-KEY ( randomized )  OR  TITLE-ABS-KEY ( random )  OR  TITLE-ABS-KEY ( randomly )  OR  TITLE-ABS-KEY ( placebo )  OR  TITLE-ABS-KEY ( assignment )  OR  TITLE-ABS-KEY ( "clinical trial" )  OR  TITLE-ABS-KEY ( trial )  OR  TITLE-ABS-KEY ( randomised ) )  **#1 and #2 and #3** |

| **Supplemental Table 2**: Reason for exclusion of retrieved articles | |
| --- | --- |
| References | Reason for exclusion |
| 1. Sleddering MA, Markvoort AJ, Dharuri HK, Jeyakar S, Snel M, Juhasz P, et al. Proteomic analysis in type 2 diabetes patients before and after a very low calorie diet reveals potential disease state and intervention specific biomarkers. PLoS ONE. 2014;9(11). | not-relevant control group |
| 1. Degoutte F, Jouanel P, Bègue R, Colombier M, Lac G, Pequignot J, Filaire E. Food restriction, performance, biochemical, psychological, and endocrine changes in jud(1)athletes. International journal of sports medicine. 2006;27(01):9-18. | Short duration of study |
| 1. Khoo J, Ling PS, Tan J, Teo A, Ng HL, Chen RY, et al. Comparing the effects of meal replacements with reduced-fat diet on weight, sexual and endothelial function, testosterone and quality of life in obese Asian men. Int J Impot Res. 2014;26(2):61-6. | not-relevant control group |
| 1. Abedelmalek S, Chtourou H, Souissi N, Tabka Z. Caloric Restriction Effect on Proinflammatory Cytokines, Growth Hormone, and Steroid Hormone Concentrations during Exercise in Judokas. Oxid Med Cell Longev. 2015;2015:809492. | Short duration of study |
| 1. Mettler S, Mitchell N, Tipton KD. Increased protein intake reduces lean body mass loss during weight loss in athletes. Med Sci Sports Exerc. 2010;42(2):326-37. | not-relevant control group |
| 1. Kok P, Roelfsema F, Langendonk JG, de Wit CC, Frölich M, Burggraaf J, et al. Increased circadian prolactin release is blunted after body weight loss in obese premenopausal women. Am J Physiol Endocrinol Metab. 2006;290(2):E218-24. | No control |
| 1. Koehler K, Hoerner NR, Gibbs JC, Zinner C, Braun H, De Souza MJ, et al. Low energy availability in exercising men is associated with reduced leptin and insulin but not with changes in other metabolic hormones. J Sports Sci. 2016;34(20):1921-9. | Short duration of study |
| 1. Winkels RM, Sturgeon KM, Kallan MJ, Dean LT, Zhang Z, Evangelisti M, et al. The women in steady exercise research (WISER) survivor trial: The innovative transdisciplinary design of a randomized controlled trial of exercise and weight-loss interventions among breast cancer survivors with lymphedema. Contemporary clinical trials. 2017;61:63-72. | Insufficient information |
| 1. Schulte DM, Hahn M, Oberhäuser F, Malchau G, Schubert M, Heppner C, et al. Caloric restriction increases serum testosterone concentrations in obese male subjects by two distinct mechanisms. Horm Metab Res. 2014;46(4):283-6. | No control |
| 1. Svendsen PF, Jensen FK, Holst JJ, Haugaard SB, Nilas L, Madsbad S. The effect of a very low calorie diet on insulin sensitivity, beta cell function, insulin clearance, incretin hormone secretion, androgen levels and body composition in obese young women. Scand J Clin Lab Invest. 2012;72(5):410 | not-relevant control group |
| 1. Huovinen HT, Hulmi JJ, Isolehto J, Kyröläinen H, Puurtinen R, Karila T, et al. Body composition and power performance improved after weight reduction in male athletes without hampering hormonal balance. The Journal of Strength & Conditioning Research. 2015;29(1):29-36. | not-relevant control group |
| 1. Wang P, Menheere PP, Astrup A, Andersen MR, van Baak MA, Larsen TM, et al. Metabolic syndrome, circulating RBP4, testosterone, and SHBG predict weight regain at 6 months after weight loss in men. Obesity. 2013;21(10):1997-2006. | No control |
| 1. Mehrabani HH, Salehpour S, Amiri Z, Farahani SJ, Meyer BJ, Tahbaz F. Beneficial effects of a high-protein, low-glycemic-load hypocaloric diet in overweight and obese women with polycystic ovary syndrome: a randomized controlled intervention study. Journal of the American College of Nutrition. 2012;31(2):117-25. | not-relevant control group |
| 1. van Gemert WA, Iestra JI, Schuit AJ, May AM, Takken T, Veldhuis WB, et al. Design of the SHAPE-2 study: the effect of physical activity, in addition to weight loss, on biomarkers of postmenopausal breast cancer risk. BMC cancer. 2013;13:1-10. | Protocol |
| 1. Harvie MN, Pegington M, Mattson MP, Frystyk J, Dillon B, Evans G, et al. The effects of intermittent or continuous energy restriction on weight loss and metabolic disease risk markers: a randomized trial in young overweight women. International journal of obesity. 2011;35(5):714-27. | not-relevant control group |
| 1. Azadi‐Yazdi M, Karimi‐Zarchi M, Salehi‐Abargouei A, Fallahzadeh H, Nadjarzadeh A: Effects of Dietary Approach to Stop Hypertension diet on androgens, antioxidant status and body composition in overweight and obese women with polycystic ovary syndrome: a randomised controlled trial. *Journal of human nutrition and dietetics* 2017, 30(3):275-283. | not-relevant control group |
| 1. Moran LJ, Brinkworth GD, Martin S, Wycherley TP, Stuckey B, Lutze J, Clifton PM, Wittert GA, Noakes M: Long-term effects of a randomised controlled trial comparing high protein or high carbohydrate weight loss diets on testosterone, SHBG, erectile and urinary function in overweight and obese men. *PloS one* 2016, 11(9):e0161297. | not-relevant control group |
| 1. Zangeneh F, Salman Yazdi R, Naghizadeh MM, Abedinia N. Effect of Ramadan Fasting on Stress Neurohormones in Women with Polycystic Ovary Syndrome. J Family Reprod Health. 2015;9(2):51-7. | Insufficient information |
| 1. Cağlayan EK, Göçmen AY, Delibas N. Effects of long-term fasting on female hormone levels: Ramadan model. Clin Exp Obstet Gynecol. 2014;41(1):17-9. | No control |
| 1. Maynard DDC, Matos RC, Damasceno IC, Brito CJ, Miarka B, Grigoletto MEDS, et al. Low versus adequate carbohydrate diet in Brazilian jiu jitsu athletes: Comparisons of hormonal biomarkers, physical and psychological. Archives of Budo. 2018;14:13-23. | No Control |
| 1. Liu H, Shangguan F, Liu F, Guo Y, Yu H, Li H, et al. Evaluating the effects of time-restricted eating on overweight and obese women with polycystic ovary syndrome: A randomized controlled trial study protocol. PLoS One. 2025;20(1):e0316333. | Insufficient information |
| 1. Shishehgar F, Mirmiran P, Rahmati M, Tohidi M, Ramezani Tehrani F. Does a restricted energy low glycemic index diet have a different effect on overweight women with or without polycystic ovary syndrome? BMC Endocr Disord. 2019;19(1):93. | No Control |
| 1. Mongioì LM, Cimino L, Condorelli RA, Magagnini MC, Barbagallo F, Cannarella R, et al. Effectiveness of a very low calorie ketogenic diet on testicular function in overweight/obese men. Nutrients. 2020;12(10):1-11. | No Control |
| 1. Stratton MT, Tinsley GM, Alesi MG, Hester GM, Olmos AA, Serafini PR, et al. Four Weeks of Time-Restricted Feeding Combined with Resistance Training Does Not Differentially Influence Measures of Body Composition, Muscle Performance, Resting Energy Expenditure, and Blood Biomarkers. Nutrients. 2020;12(4). | not-relevant control group |
| 1. Tabrizi FPF, Farhangi MA, Vaezi M, Hemmati S. The effects of spinach-derived thylakoid supplementation in combination with calorie restriction on anthropometric parameters and metabolic profiles in obese women with polycystic ovary syndrome: a randomized, double-blind, placebo-controlled clinical trial. Nutr J. 2020;19(1):82. | No Control |
| 1. Li C, Xing C, Zhang J, Zhao H, Shi W, He B. Eight-hour time-restricted feeding improves endocrine and metabolic profiles in women with anovulatory polycystic ovary syndrome. J Transl Med. 2021;19(1):148. | No Control |
| 1. Peos JJ, Helms ER, Fournier PA, Ong J, Hall C, Krieger J, et al. Continuous versus Intermittent Dieting for Fat Loss and Fat-Free Mass Retention in Resistance-trained Adults: The ICECAP Trial. Med Sci Sports Exerc. 2021;53(8):1685-98. | No Control |
| 1. Deshmukh H, Papageorgiou M, Wells L, Akbar S, Strudwick T, Deshmukh K, et al. The Effect of a Very-Low-Calorie Diet (VLCD) vs. a Moderate Energy Deficit Diet in Obese Women with Polycystic Ovary Syndrome (PCOS)—A Randomised Controlled Trial. Nutrients. 2023;15(18). | not-relevant control group |
| 1. Feyzioglu BS, Güven CM, Avul Z. Eight-Hour Time-Restricted Feeding: A Strong Candidate Diet Protocol for First-Line Therapy in Polycystic Ovary Syndrome. Nutrients. 2023;15(10). | No Control |
| 1. 11. Guerrieri-Gonzaga A, Serrano D, Gnagnarella P, Johansson H, Zovato S, Nardi M, et al. Low dose TamOxifen and LifestylE changes for bReast cANcer prevention (TOLERANT study): Study protocol of a randomized phase II biomarker trial in women at increased risk for breast cancer. PLoS One. 2024;19(9):e0309511. | Insufficient information |
| 1. Moini A, Arabipoor A, Hemat M, Ahmadi J, Salman-Yazdi R, Zolfaghari Z. The effect of weight loss program on serum anti-Müllerian hormone level in obese and overweight infertile women with polycystic ovary syndrome. Gynecol Endocrinol. 2019;35(2):119-23. | No Control |
| 1. Dou P, Zhang T, Xu Y, Xue Q, Shang J, Yang X. Effects of three medical nutrition therapies for weight loss on metabolic parameters and androgen level in overweight/obese patients with polycystic ovary syndrome. Zhonghua yi xue za zhi. 2023;103(14):1035-41. | not-relevant control group |
| 1. Jurov I, Keay N, Rauter S. Reducing energy availability in male endurance athletes: a randomized trial with a three-step energy reduction. Journal of the International Society of Sports Nutrition. 2022;19(1):179-95. | Insufficient information |
| 1. Wang P, Menheere PP, Astrup A, Andersen MR, van Baak MA, Larsen TM, et al. Metabolic syndrome, circulating RBP4, testosterone, and SHBG predict weight regain at 6 months after weight loss in men. Obesity. 2013;21(10):1997-2006. | No control |
| 1. Szczuko M, Zapalowska-Chwyć M, Drozd R: A low glycemic index decreases inflammation by increasing the concentration of uric acid and the activity of glutathione peroxidase (GPx3) in patients with polycystic ovary syndrome (PCOS). *Molecules* 2019, 24(8):1508. | not-relevant control group |
| 1. Vidić V, Ilić V, Toskić L, Janković N, Ugarković D: Effects of calorie restricted low carbohydrate high fat ketogenic vs. non-ketogenic diet on strength, body-composition, hormonal and lipid profile in trained middle-aged men. *Clinical Nutrition* 2021, 40(4):1495-1502. | not-relevant control group |

| Supplementary table 3. Subgroup analysis to evaluate the effect of all types of fasting diet and calorie restricted diet on reproductive related hormones | | | | | |
| --- | --- | --- | --- | --- | --- |
| Sub grouped by | No. | WMD (95% CI) | P-value | P-Heterogeneity | I^2^ (%) |
| Testosterone (Fasting) | | | | | |
| Location | | | | | |
| USA | 3 | -0.05 (-0.10, 0.002) | 0.06 | 0.00 | 99.3 |
| Non- USA | 2 | -1.39 (-1.90, -0.89) | 0.00 | 0.65 | 0.0 |
| Testosterone (Calorie restricted) | | | | | |
| Duration | | | | | |
| <16 weeks | 3 | 0.005 (-0.02, 0.03) | 0.76 | 0.00 | 96.3 |
| >=16 weeks | 8 | -0.000 (-0.004, 0.003) | 0.85 | 0.00 | 95.8 |
| Year of publication | | | | | |
| <2015 | 3 | -0.04 (-0.10, 0.02) | 0.21 | 0.09 | 58.1 |
| >=2015 | 8 | -0.000 (-0.004, 0.004) | 0.94 | 0.00 | 96.8 |
| Location | | | | | |
| USA | 5 | -0.02 (-0.06, 0.01) | 0.22 | 0.00 | 97.5 |
| Non- USA | 6 | -0.000 (-0.004, 0.004) | 0.97 | 0.00 | 91.7 |
| Sample size | | | | | |
| <50 | 8 | -0.02 (-0.05, 0.01) | 0.17 | 0.00 | 96.8 |
| >=50 | 3 | -0.000 (-0.004, 0.004) | 0.99 | 0.34 | 7 |
| Free Testosterone (Calorie restricted) | | | | | |
| Duration | | | | | |
| <16 weeks | 2 | 0.49 (0.21, 0.76) | 0.00 | 0.00 | 98.9 |
| >=16 weeks | 4 | -1.21 (-2.12, -0.31) | 0.008 | 0.02 | 68.3 |
| Year of publication | | | | | |
| <2015 | 2 | 5.33 (3.79, 6.87) | 0.00 | 0.00 | 98.4 |
| >=2015 | 4 | 0.19 (-0.06, 0.46) | 0.14 | 0.00 | 74.2 |
| Location | | | | | |
| USA | 2 | -0.97 (-1.97, 0.02) | 0.056 | 0.01 | 83 |
| Non- USA | 4 | 0.44 (0.17, 0.71) | 0.00 | 0.00 | 97.1 |
| Sample size | | | | | |
| <50 | 3 | 1.03 (0.18, 1.87) | 0.01 | 0.00 | 98.1 |
| >=50 | 3 | 0.27 (-0.002, 0.54) | 0.052 | 0.02 | 73.3 |
| SHBG (Fasting) | | | | | |
| Duration | | | | | |
| <16 weeks | 1 | 42.75 (-460.37, 545.87) | 0.86 | 0.00 | 92.2 |
| >=16 weeks | 3 | 49.47 (34.14, 64.80) | 0.00 | 0.00 | 88.2 |
| Location | | | | | |
| USA | 3 | 49.47 (34.14, 64.80) | 0.00 | 0.00 | 92.2 |
| Non- USA | 1 | 42.75 (-460.37, 545.87) | 0.86 | - | - |
| SHBG (Calorie restricted) | | | | | |
| Duration | | | | | |
| <16 weeks | 2 | -540.45 (-130.78,239.64) | 0.17 | 0.02 | 81.4 |
| >=16 weeks | 8 | -69.78 (-81.42, -58.13) | 0.00 | 0.00 | 90.1 |
| Year of publication | | | | | |
| <2015 | 3 | -116.19 (-403.97,171.58) | 0.42 | 0.02 | 71.7 |
| >=2015 | 7 | -69.81 (-81.464, -58.15) | 0.00 | 0.00 | 91.5 |
| Location | | | | | |
| USA | 5 | -69.72 (-81.38, -58.07) | 0.00 | 0.00 | 93.9 |
| Non- USA | 5 | -152.00(-416.28, 112.27) | 0.26 | 0.01 | 66.8 |
| Sample size | | | | | |
| <50 | 7 | -71.56 (-83.21, -59.90) | 0.00 | 0.02 | 58.3 |
| >=50 | 3 | 856.29 (582.04,1130.54) | 0.00 | 0.00 | 89.8 |
| LH (Calorie restricted) | | | | | |
| Duration | | | | | |
| <16 weeks | 1 | -1.780 (-3.27, -0.28) | 0.01 | - | - |
| >=16 weeks | 2 | 0.58 (0.23, 0.92) | 0.001 | 0.68 | 0.0 |
| Year of publication | | | | | |
| <2015 | 1 | 0.80 (-0.31, 1.91) | 0.16 | - | - |
| >=2015 | 2 | 0.46 (0.12, 0.79) | 0.01 | 0.003 | 88.8 |
| Location | | | | | |
| USA | 1 | 0.56 (0.19, 0.92) | 0.003 | - | - |
| Non- USA | 2 | -0.12 (-1.02, 0.76) | 0.78 | 0.007 | 86.4 |
| Sample size | | | | | |
| <50 | 1 | 0.80 (-0.31, 1.91) | 0.16 | - | - |
| >=50 | 2 | 0.42 (0.07, 0.78) | 0.01 | 0.003 | 88.8 |
| FSH (Calorie restricted) | | | | | |
| Duration | | | | | |
| <16 weeks | 1 | -0.03 (-0.38, 0.32) | 0.86 | - | - |
| >=16 weeks | 2 | 0.34 (-0.19, 0.88) | 0.20 | 0.15 | 50.4 |
| Year of publication | | | | | |
| <2015 | 1 | 0.10 (-0.54, 0.74) | 0.75 | - | - |
| >=2015 | 2 | 0.07 (-0.25, 0.41) | 0.64 | 0.06 | 69.8 |
| Location | | | | | |
| USA | 1 | 0.97 (-0.04, 1.98) | 0.06 | - | - |
| Non- USA | 2 | 0.08 (-0.21, 0.38) | 0.99 | 0.72 | 0.0 |

**Supplementary Table 4.** Risk of bias of included studies.

| **Publications** | **Randomization process** | **Deviations from the intended interventions** | **Missing outcome data** | **Measurement of the outcome** | **Selection of the reported result** | **Overall Bias** |
| --- | --- | --- | --- | --- | --- | --- |
| Kaukua (2003), Finland | S | H | L | L | S | H |
| Villareal (2006), USA | L | H | L | L | L | H |
| Heilbronn (2006), USA | L | S | L | L | L | L |
| Khoo (2011), Australia | S | H | H | L | L | H |
| Nybacka (2013), Sweden | L | H | S | L | L | H |
| Van Gemert (2015), Netherland | L | S | L | L | L | L |
| Pop (2015), USA | S | H | S | L | L | H |
| Moro (2016), Italy | L | H | L | L | S | H |
| Martin (2016), USA | L | L | S | L | L | L |
| Moro (2020), Italy | L | H | L | L | S | H |
| Moro (2021), Italy | L | H | L | L | S | H |
| Kulshreshtha (2023), India | L | H | H | L | S | H |
| Sampieri (2024), Italy | L | H | L | L | L | H |
| Lin (2024), USA | L | H | S | L | L | H |
| Garcia-Morales (2025), Spain | S | H | L | L | L | H |

**Supplementary Table 5**. GRADE evidence table for the effects of fasting diet and calorie restricted diet on reproductive related hormones.

| **Certainty assessment** | | | | | | | **№ of patients** | | **Effect** | | **Certainty** | **Importance** |
| --- | --- | --- | --- | --- | --- | --- | --- | --- | --- | --- | --- | --- |
| **№ of studies** | **Study design** | **Risk of bias** | **Inconsistency** | **Indirectness** | **Imprecision** | **Other considerations** | **[intervention]** | **[comparison]** | **Relative (95% CI)** | **Absolute (95% CI)** |  |  |
| **Testosterone (Studies on fasting diet) (follow-up: range 4 weeks to 96 weeks)** | | | | | | | | | | | | |
| 3 | randomised trials | serious^a^ | very serious^b^ | not serious | serious^c^ | none | 63 | 64 | - | **0**  (0 to 0 ) | ⨁◯◯◯ Very low^a,b,c^ | IMPORTANT |
| **Testosterone (Studies on calorie restricted diet) (follow-up: range 4 weeks to 96 weeks)** | | | | | | | | | | | | |
| 9 | randomised trials | serious^a^ | very serious^d^ | not serious | serious^c^ | none | 415 | 301 | - | **0**  (0 to 0 ) | ⨁◯◯◯ Very low^a,c,d^ | IMPORTANT |
| **Free Testosterone (Studies on fasting diet) (follow-up: range 4 weeks to 96 weeks)** | | | | | | | | | | | | |
| 2 | randomised trials | serious^a^ | not serious | not serious | not serious | none | 38 | 38 | - | **0**  (0 to 0 ) | ⨁⨁⨁◯ Moderate^a^ | IMPORTANT |
| **Free Testosterone (Studies on calorie restricted diet) (follow-up: range 4 weeks to 96 weeks)** | | | | | | | | | | | | |
| 6 | randomised trials | serious^a^ | very serious^e^ | not serious | serious^c^ | none | 358 | 241 | - | **0**  (0 to 0 ) | ⨁◯◯◯ Very low^a,c,e^ | IMPORTANT |
| **SHBG (Studies on fasting diet) (follow-up: range 4 weeks to 96 weeks)** | | | | | | | | | | | | |
| 2 | randomised trials | serious^a^ | serious^f^ | not serious | serious^c^ | none | 44 | 45 | - | **0**  (0 to 0 ) | ⨁◯◯◯ Very low^a,c,f^ | IMPORTANT |
| **SHBG (Studies on calorie restricted diet) (follow-up: range 4 weeks to 96 weeks)** | | | | | | | | | | | | |
| 8 | randomised trials | serious^a^ | serious^g^ | not serious | serious^c^ | none | 403 | 285 | - | **0**  (0 to 0 ) | ⨁◯◯◯ Very low^a,c,g^ | IMPORTANT |
| **DHEA-S (Studies on fasting diet) (follow-up: range 4 weeks to 96 weeks)** | | | | | | | | | | | | |
| 1 | randomised trials | serious^a^ | serious^h^ | not serious | serious^c^ | none | 36 | 37 | - | **0**  (0 to 0 ) | ⨁◯◯◯ Very low^a,c,h^ | IMPORTANT |
| **DHEA-S (Studies on calorie restricted diet) (follow-up: range 4 weeks to 96 weeks)** | | | | | | | | | | | | |
| 2 | randomised trials | serious^a^ | serious^i^ | not serious | serious^c^ | none | 48 | 49 | - | **0**  (0 to 0 ) | ⨁◯◯◯ Very low^a,c,i^ | IMPORTANT |
| **LH (Studies on calorie restricted diet) (follow-up: range 4 weeks to 96 weeks)** | | | | | | | | | | | | |
| 3 | randomised trials | serious^a^ | serious^j^ | not serious | serious^c^ | none | 224 | 157 | - | **0**  (0 to 0 ) | ⨁◯◯◯ Very low^a,c,j^ | IMPORTANT |
| **FSH (Studies on calorie restricted diet) (follow-up: range 4 weeks to 96 weeks)** | | | | | | | | | | | | |
| 3 | randomised trials | serious^a^ | not serious | not serious | serious^c^ | none | 224 | 157 | - | **0**  (0 to 0 ) | ⨁⨁◯◯ Low^a,c^ | IMPORTANT |
| **Estradiol (Studies on calorie restricted diet) (follow-up: range 4 weeks to 96 weeks)** | | | | | | | | | | | | |
| 4 | randomised trials | serious^a^ | serious^k^ | not serious | serious^c^ | none | 143 | 62 | - | **0**  (0 to 0 ) | ⨁◯◯◯ Very low^a,c,k^ | IMPORTANT |

**CI:** confidence interval; **MD:** mean difference

#### Explanations

a. Serious risk of bias since >50% studies had high risk of bias

b. Serious inconsistency since I2 = 98.8%, P-het <0.001. Downgraded

c. Based on null effect approach.

d. Serious inconsistency since I2 = 95.5%, P-het <0.001. Downgraded.

e. Serious inconsistency since I2 = 95.7%, P-het <0.001. Downgraded.

f. Serious inconsistency since I2 = 88.2%, P-het <0.001. Downgraded.

g. Serious inconsistency since I2 = 88.4%, P-het <0.001. Downgraded.

h. Serious inconsistency since I2 = 90.4%, P-het <0.001. Downgraded.

i. Serious inconsistency since I2 = 75.9%, P-het <0.001. Downgraded.

j. Serious inconsistency since I2 = 78.5%, P-het <0.001. Downgraded.

k. Serious inconsistency since I2 = 97.8%, P-het <0.001. Downgraded.
